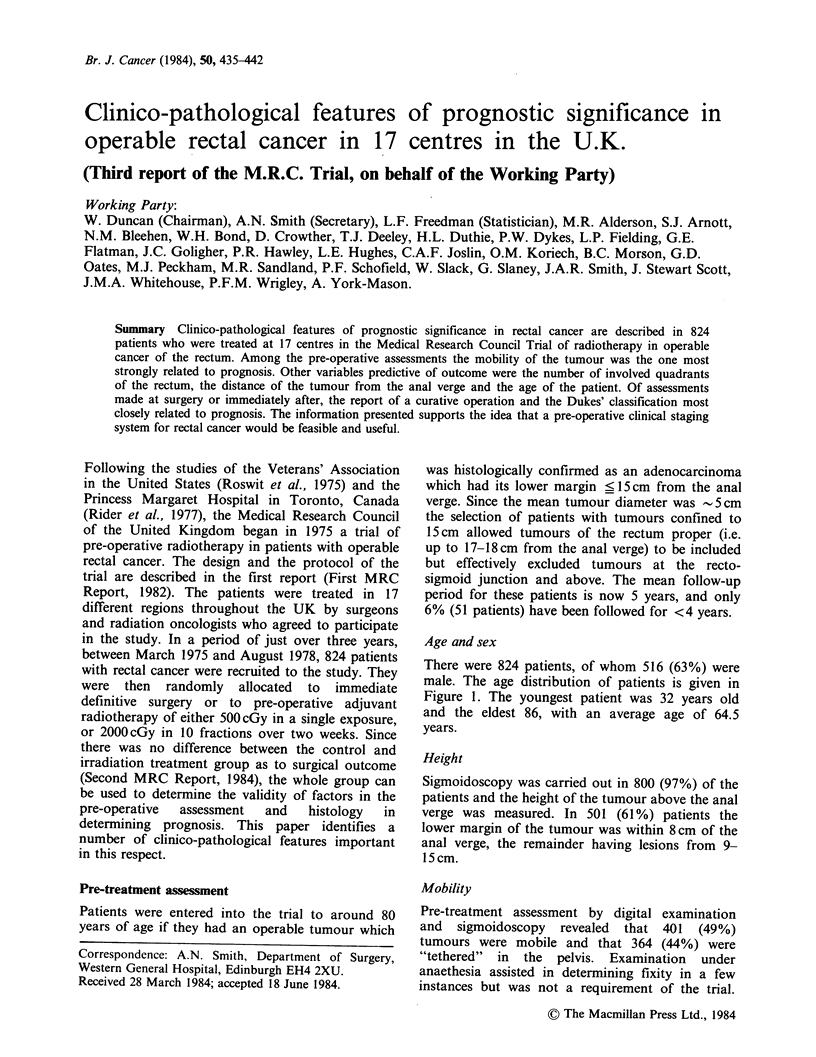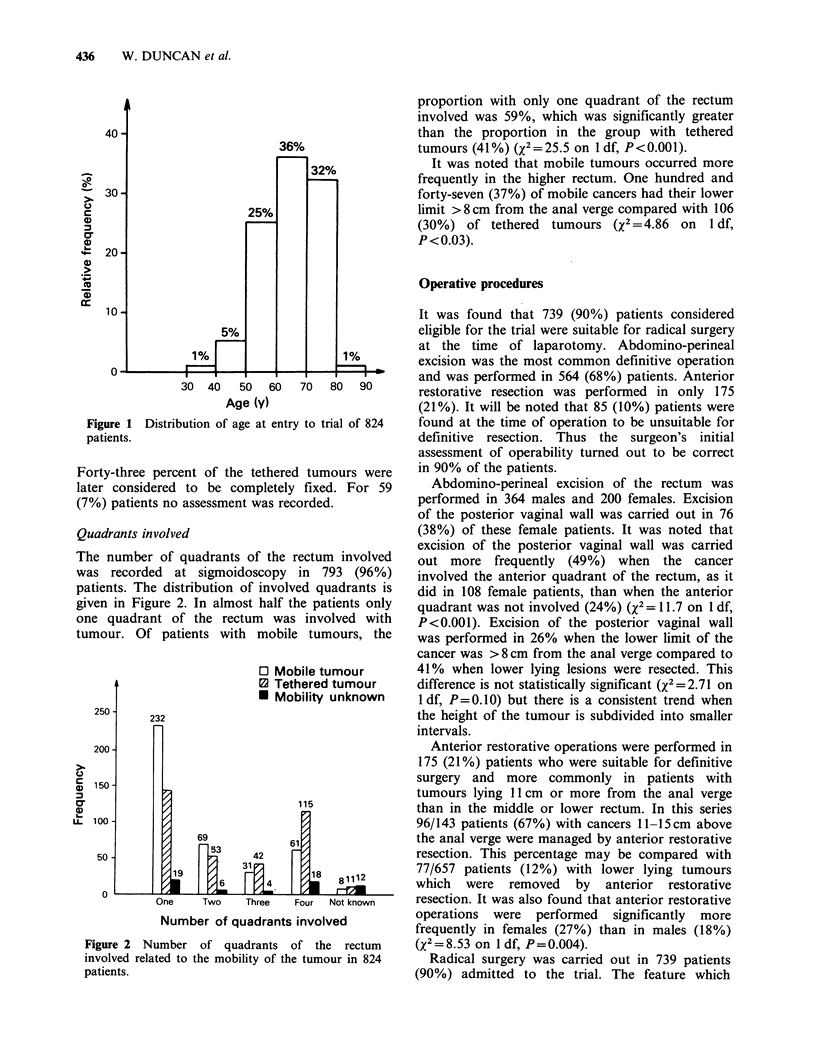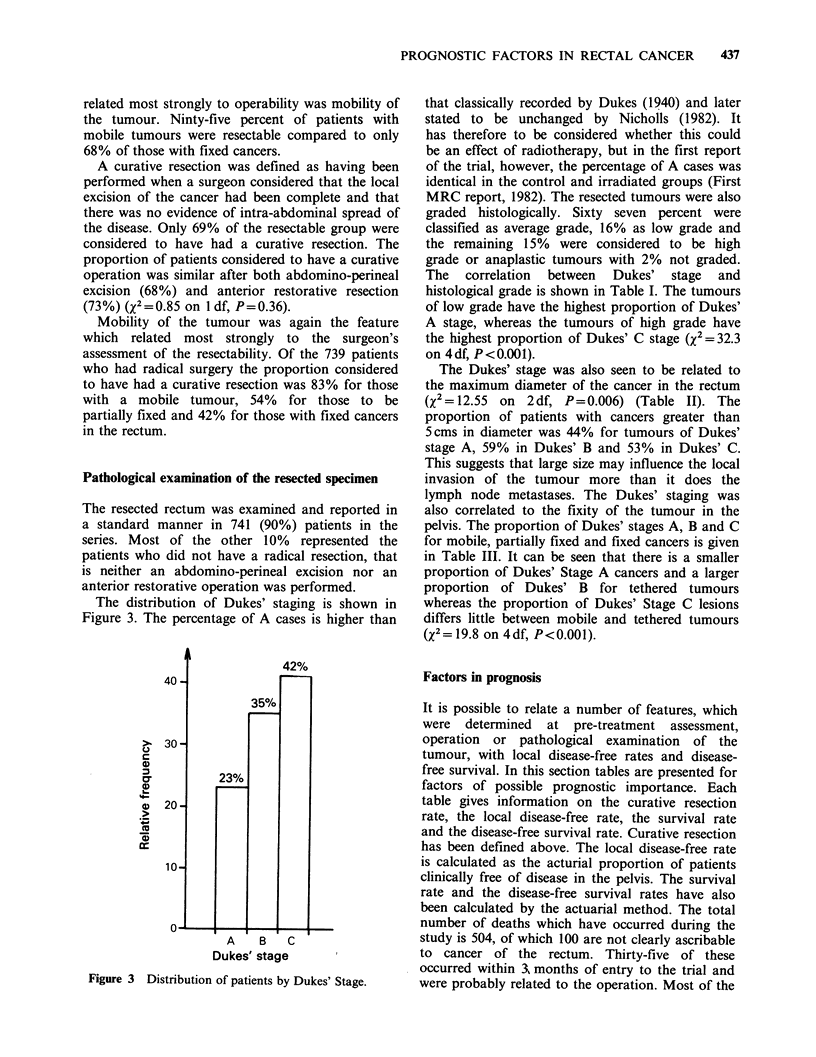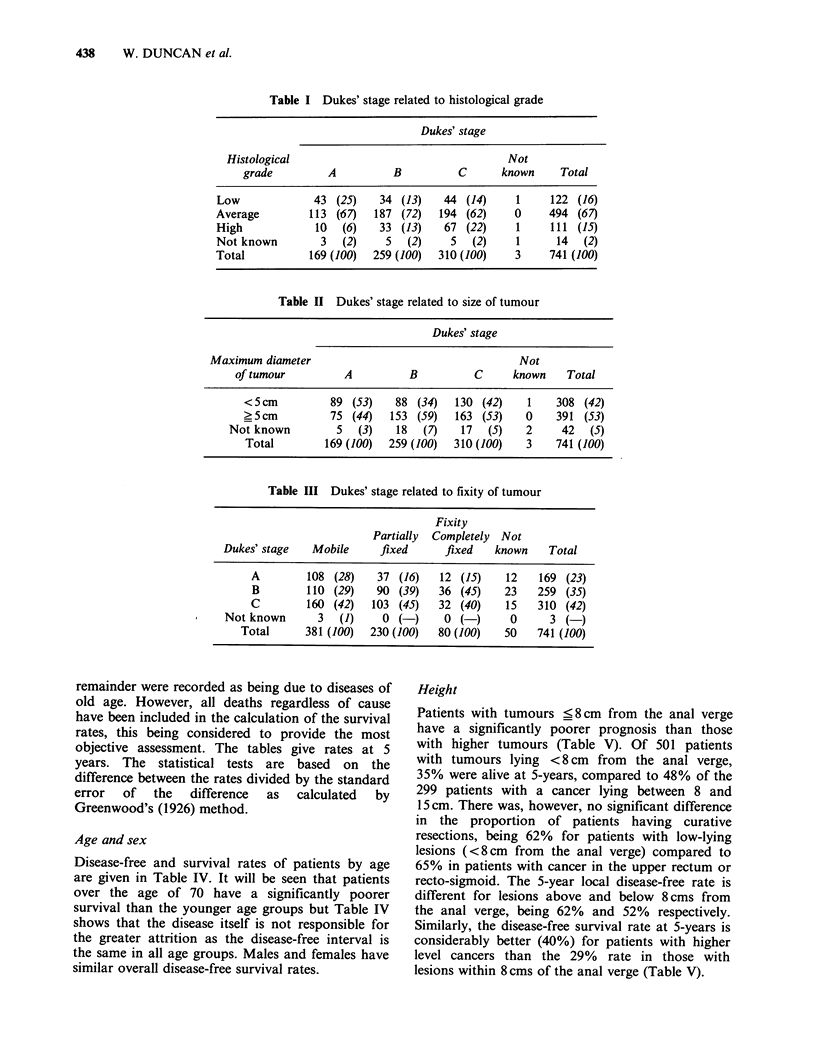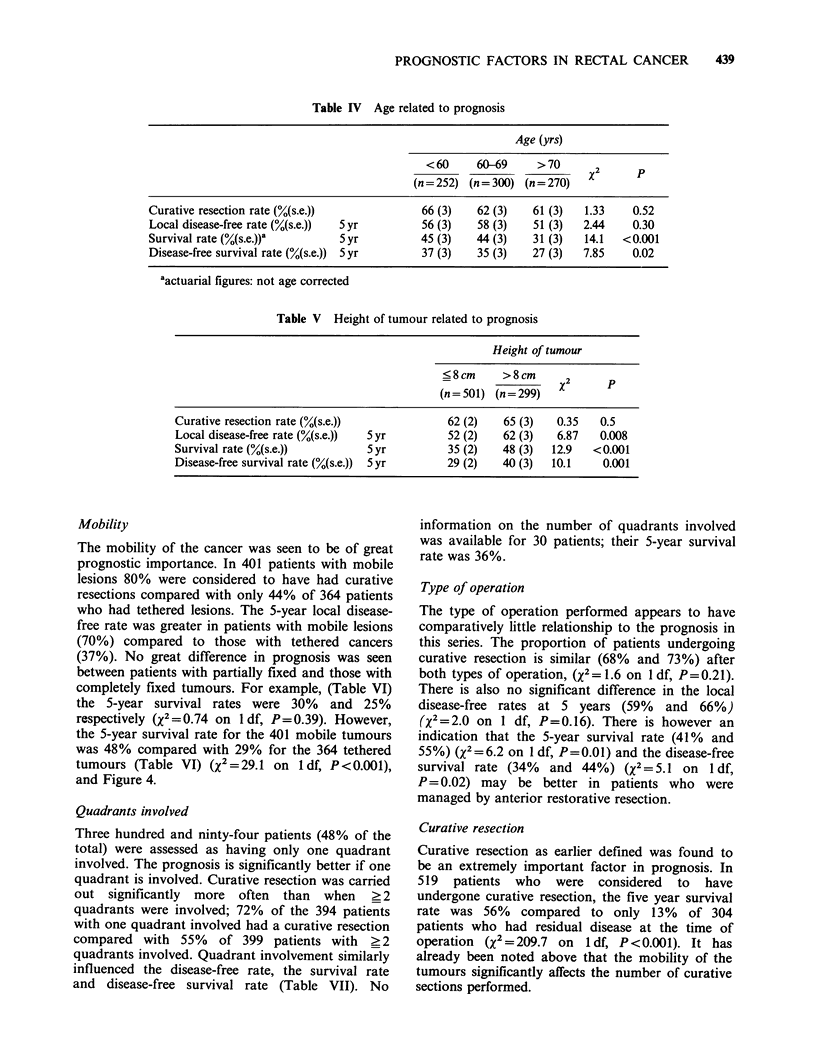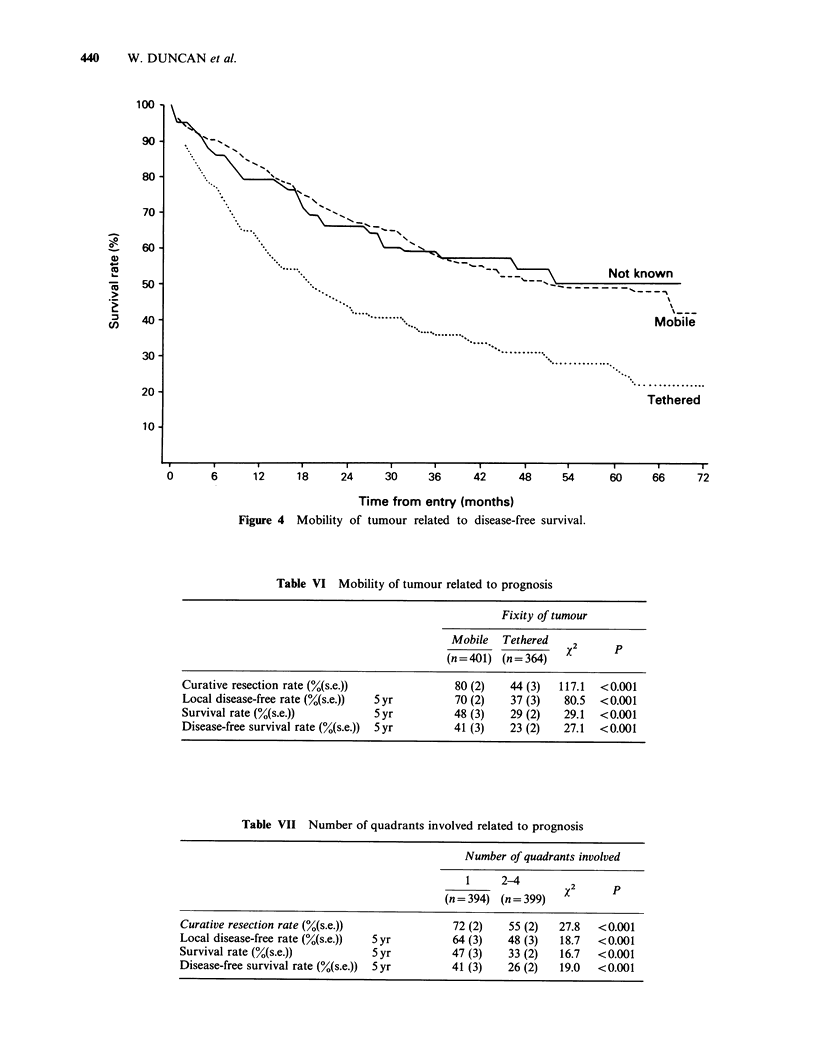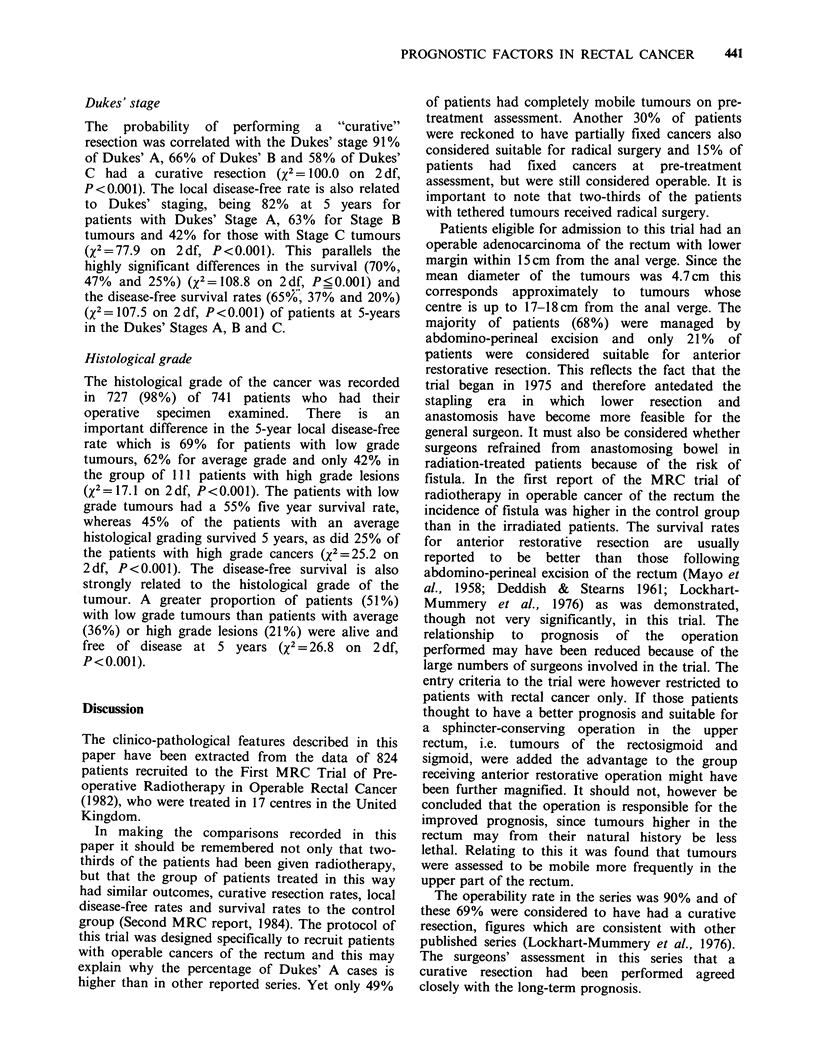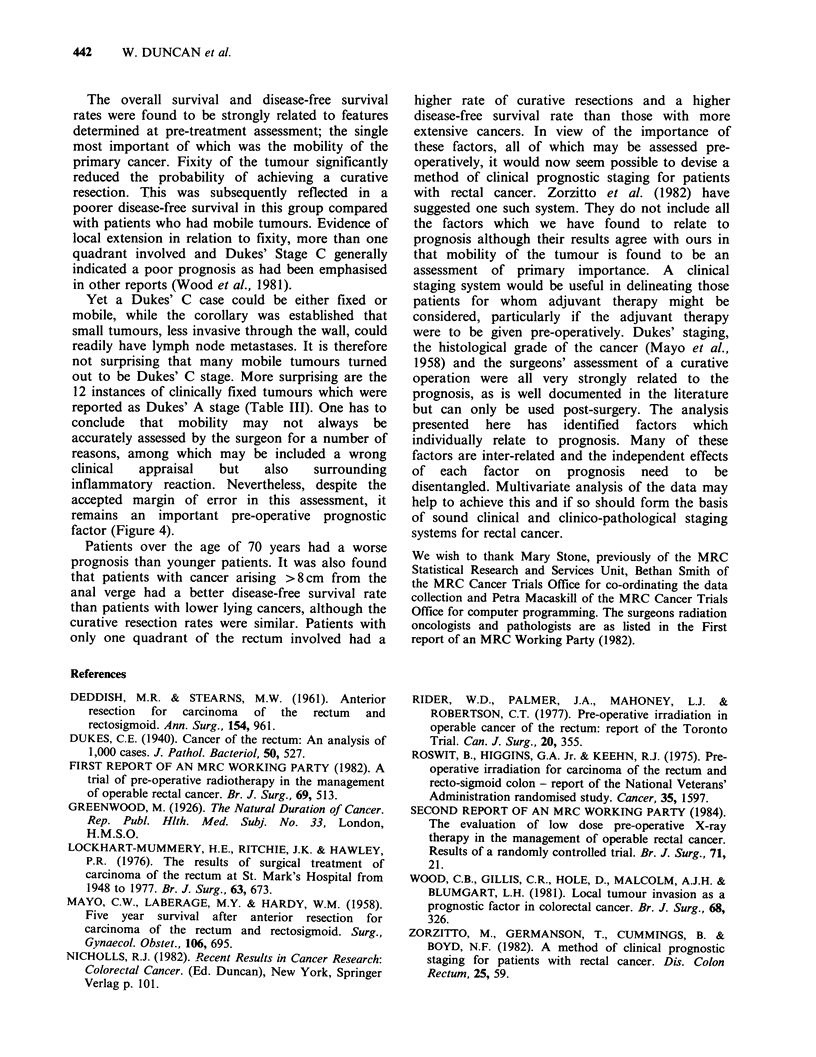# Clinico-pathological features of prognostic significance in operable rectal cancer in 17 centres in the U.K. (Third report of the M.R.C. Trial, on behalf of the Working Party).

**DOI:** 10.1038/bjc.1984.198

**Published:** 1984-10

**Authors:** 

## Abstract

Clinico-pathological features of prognostic significance in rectal cancer are described in 824 patients who were treated at 17 centres in the Medical Research Council Trial of radiotherapy in operable cancer of the rectum. Among the pre-operative assessments the mobility of the tumour was the one most strongly related to prognosis. Other variables predictive of outcome were the number of involved quadrants of the rectum, the distance of the tumour from the anal verge and the age of the patient. Of assessments made at surgery or immediately after, the report of a curative operation and the Dukes' classification most closely related to prognosis. The information presented supports the idea that a pre-operative clinical staging system for rectal cancer would be feasible and useful.


*Br. J. Cancer* (1984), **50**, 435–442

### Clinico-pathological features of prognostic significance in operable rectal cancer in 17 centres in the U.K.

### (Third report of the M.R.C. Trial, on behalf of the Working Party)

*Working Party*:
W. Duncan (Chairman), A.N. Smith (Secretary), L.F. Freedman (Statistician), M.R. Alderson, S.J. Arnott, N.M. Bleehen, W.H. Bond, D. Crowther, T.J. Deeley, H.L. Duthie, P.W. Dykes, L.P. Fielding, G.E. Flatman, J.C. Goligher, P.R. Hawley, L.E. Hughes, C.A.F. Joslin, O.M. Koriech, B.C. Morson, G.D. Oates, M.J. Peckham, M.R. Sandland, P.F. Schofield, W. Slack, G. Slaney, J.A.R. Smith, J. Stewart Scott, J.M.A. Whitehouse, P.F.M. Wrigley, A. York-Mason.

**Summary** Clinico-pathological features of prognostic significance in rectal cancer are described in 824 patients who were treated at 17 centres in the Medical Research Council Trial of radiotherapy in operable cancer of the rectum. Among the pre-operative assessments the mobility of the tumour was the one most strongly related to prognosis. Other variables predictive of outcome were the number of involved quadrants of the rectum, the distance of the tumour from the anal verge and the age of the patient. Of assessments made at surgery or immediately after, the report of a curative operation and the Dukes' classification most closely related to prognosis. The information presented supports the idea that a pre-operative clinical staging system for rectal cancer would be feasible and useful.

Following the studies of the Veterans' Association in the United States (Roswit *et al.*, 1975) and the Princess Margaret Hospital in Toronto, Canada (Rider *et al.*, 1977), the Medical Research Council of the United Kingdom began in 1975 a trial of pre-operative radiotherapy in patients with operable rectal cancer. The design and the protocol of the trial are described in the first report (First MRC Report, 1982). The patients were treated in 17 different regions throughout the UK by surgeons and radiation oncologists who agreed to participate in the study. In a period of just over three years, between March 1975 and August 1978, 824 patients with rectal cancer were recruited to the study. They were then randomly allocated to immediate definitive surgery or to pre-operative adjuvant radiotherapy of either 500 cGy in a single exposure, or 2000 cGy in 10 fractions over two weeks. Since there was no difference between the control and irradiation treatment group as to surgical outcome (Second MRC Report, 1984), the whole group can be used to determine the validity of factors in the pre-operative assessment and histology in determining prognosis. This paper identifies a number of clinico-pathological features important in this respect.

### Pre-treatment assessment

Patients were entered into the trial to around 80 years of age if they had an operable tumour which was histologically confirmed as an adenocarcinoma which had its lower margin $\leq 15$ cm from the anal verge. Since the mean tumour diameter was $\sim 5$ cm the selection of patients with tumours confined to 15 cm allowed tumours of the rectum proper (i.e. up to 17–18 cm from the anal verge) to be included but effectively excluded tumours at the recto-sigmoid junction and above. The mean follow-up period for these patients is now 5 years, and only 6% (51 patients) have been followed for $<4$ years.

### *Age and sex*

There were 824 patients, of whom 516 (63%) were male. The age distribution of patients is given in Figure 1. The youngest patient was 32 years old and the eldest 86, with an average age of 64.5 years.

### *Height*

Sigmoidoscopy was carried out in 800 (97%) of the patients and the height of the tumour above the anal verge was measured. In 501 (61%) patients the lower margin of the tumour was within 8 cm of the anal verge, the remainder having lesions from 9–15 cm.

### *Mobility*

Pre-treatment assessment by digital examination and sigmoidoscopy revealed that 401 (49%) tumours were mobile and that 364 (44%) were "tethered" in the pelvis. Examination under anaethesia assisted in determining fixity in a few instances but was not a requirement of the trial.

Correspondence: A.N. Smith, Department of Surgery, Western General Hospital, Edinburgh EH4 2XU.
Received 28 March 1984; accepted 18 June 1984.

© The Macmillan Press Ltd., 1984

**Figure 1** Distribution of age at entry to trial of 824 patients.

Forty-three percent of the tethered tumours were later considered to be completely fixed. For 59 (7%) patients no assessment was recorded.

*Quadrants involved*

The number of quadrants of the rectum involved was recorded at sigmoidoscopy in 793 (96%) patients. The distribution of involved quadrants is given in Figure 2. In almost half the patients only one quadrant of the rectum was involved with tumour. Of patients with mobile tumours, the

**Figure 2** Number of quadrants of the rectum involved related to the mobility of the tumour in 824 patients.

proportion with only one quadrant of the rectum involved was 59%, which was significantly greater than the proportion in the group with tethered tumours (41%) ($\chi^2=25.5$ on 1 df, $P<0.001$).

It was noted that mobile tumours occurred more frequently in the higher rectum. One hundred and forty-seven (37%) of mobile cancers had their lower limit >8 cm from the anal verge compared with 106 (30%) of tethered tumours ($\chi^2=4.86$ on 1 df, $P<0.03$).

### Operative procedures

It was found that 739 (90%) patients considered eligible for the trial were suitable for radical surgery at the time of laparotomy. Abdomino-perineal excision was the most common definitive operation and was performed in 564 (68%) patients. Anterior restorative resection was performed in only 175 (21%). It will be noted that 85 (10%) patients were found at the time of operation to be unsuitable for definitive resection. Thus the surgeon's initial assessment of operability turned out to be correct in 90% of the patients.

Abdomino-perineal excision of the rectum was performed in 364 males and 200 females. Excision of the posterior vaginal wall was carried out in 76 (38%) of these female patients. It was noted that excision of the posterior vaginal wall was carried out more frequently (49%) when the cancer involved the anterior quadrant of the rectum, as it did in 108 female patients, than when the anterior quadrant was not involved (24%) ($\chi^2=11.7$ on 1 df, $P<0.001$). Excision of the posterior vaginal wall was performed in 26% when the lower limit of the cancer was >8 cm from the anal verge compared to 41% when lower lying lesions were resected. This difference is not statistically significant ($\chi^2=2.71$ on 1 df, $P=0.10$) but there is a consistent trend when the height of the tumour is subdivided into smaller intervals.

Anterior restorative operations were performed in 175 (21%) patients who were suitable for definitive surgery and more commonly in patients with tumours lying 11 cm or more from the anal verge than in the middle or lower rectum. In this series 96/143 patients (67%) with cancers 11–15 cm above the anal verge were managed by anterior restorative resection. This percentage may be compared with 77/657 patients (12%) with lower lying tumours which were removed by anterior restorative resection. It was also found that anterior restorative operations were performed significantly more frequently in females (27%) than in males (18%) ($\chi^2=8.53$ on 1 df, $P=0.004$).

Radical surgery was carried out in 739 patients (90%) admitted to the trial. The feature which related most strongly to operability was mobility of the tumour. Ninty-five percent of patients with mobile tumours were resectable compared to only 68% of those with fixed cancers.

A curative resection was defined as having been performed when a surgeon considered that the local excision of the cancer had been complete and that there was no evidence of intra-abdominal spread of the disease. Only 69% of the resectable group were considered to have had a curative resection. The proportion of patients considered to have a curative operation was similar after both abdomino-perineal excision (68%) and anterior restorative resection (73%) ($\chi^2=0.85$ on 1 df, $P=0.36$).

Mobility of the tumour was again the feature which related most strongly to the surgeon's assessment of the resectability. Of the 739 patients who had radical surgery the proportion considered to have had a curative resection was 83% for those with a mobile tumour, 54% for those to be partially fixed and 42% for those with fixed cancers in the rectum.

### Pathological examination of the resected specimen

The resected rectum was examined and reported in a standard manner in 741 (90%) patients in the series. Most of the other 10% represented the patients who did not have a radical resection, that is neither an abdomino-perineal excision nor an anterior restorative operation was performed.

The distribution of Dukes' staging is shown in Figure 3. The percentage of A cases is higher than

**Figure 3** Distribution of patients by Dukes' Stage.

that classically recorded by Dukes (1940) and later stated to be unchanged by Nicholls (1982). It has therefore to be considered whether this could be an effect of radiotherapy, but in the first report of the trial, however, the percentage of A cases was identical in the control and irradiated groups (First MRC report, 1982). The resected tumours were also graded histologically. Sixty seven percent were classified as average grade, 16% as low grade and the remaining 15% were considered to be high grade or anaplastic tumours with 2% not graded. The correlation between Dukes' stage and histological grade is shown in Table I. The tumours of low grade have the highest proportion of Dukes' A stage, whereas the tumours of high grade have the highest proportion of Dukes' C stage ($\chi^2=32.3$ on 4 df, $P<0.001$).

The Dukes' stage was also seen to be related to the maximum diameter of the cancer in the rectum ($\chi^2=12.55$ on 2 df, $P=0.006$) (Table II). The proportion of patients with cancers greater than 5 cms in diameter was 44% for tumours of Dukes' stage A, 59% in Dukes' B and 53% in Dukes' C. This suggests that large size may influence the local invasion of the tumour more than it does the lymph node metastases. The Dukes' staging was also correlated to the fixity of the tumour in the pelvis. The proportion of Dukes' stages A, B and C for mobile, partially fixed and fixed cancers is given in Table III. It can be seen that there is a smaller proportion of Dukes' Stage A cancers and a larger proportion of Dukes' B for tethered tumours whereas the proportion of Dukes' Stage C lesions differs little between mobile and tethered tumours ($\chi^2=19.8$ on 4 df, $P<0.001$).

### Factors in prognosis

It is possible to relate a number of features, which were determined at pre-treatment assessment, operation or pathological examination of the tumour, with local disease-free rates and disease-free survival. In this section tables are presented for factors of possible prognostic importance. Each table gives information on the curative resection rate, the local disease-free rate, the survival rate and the disease-free survival rate. Curative resection has been defined above. The local disease-free rate is calculated as the acturial proportion of patients clinically free of disease in the pelvis. The survival rate and the disease-free survival rates have also been calculated by the actuarial method. The total number of deaths which have occurred during the study is 504, of which 100 are not clearly ascribable to cancer of the rectum. Thirty-five of these occurred within 3 months of entry to the trial and were probably related to the operation. Most of the

**Table I** Dukes' stage related to histological grade

| *Histological grade* | *Dukes' stage* A | B | C | Not known | Total |
|---|---|---|---|---|---|
| Low | 43 (*25*) | 34 (*13*) | 44 (*14*) | 1 | 122 (*16*) |
| Average | 113 (*67*) | 187 (*72*) | 194 (*62*) | 0 | 494 (*67*) |
| High | 10 (*6*) | 33 (*13*) | 67 (*22*) | 1 | 111 (*15*) |
| Not known | 3 (*2*) | 5 (*2*) | 5 (*2*) | 1 | 14 (*2*) |
| Total | 169 (*100*) | 259 (*100*) | 310 (*100*) | 3 | 741 (*100*) |

**Table II** Dukes' stage related to size of tumour

| *Maximum diameter of tumour* | *Dukes' stage* A | B | C | Not known | Total |
|---|---|---|---|---|---|
| <5 cm | 89 (*53*) | 88 (*34*) | 130 (*42*) | 1 | 308 (*42*) |
| ≧5 cm | 75 (*44*) | 153 (*59*) | 163 (*53*) | 0 | 391 (*53*) |
| Not known | 5 (*3*) | 18 (*7*) | 17 (*5*) | 2 | 42 (*5*) |
| Total | 169 (*100*) | 259 (*100*) | 310 (*100*) | 3 | 741 (*100*) |

**Table III** Dukes' stage related to fixity of tumour

| *Dukes' stage* | *Fixity* Mobile | Partially fixed | Completely fixed | Not known | Total |
|---|---|---|---|---|---|
| A | 108 (*28*) | 37 (*16*) | 12 (*15*) | 12 | 169 (*23*) |
| B | 110 (*29*) | 90 (*39*) | 36 (*45*) | 23 | 259 (*35*) |
| C | 160 (*42*) | 103 (*45*) | 32 (*40*) | 15 | 310 (*42*) |
| Not known | 3 (*1*) | 0 (—) | 0 (—) | 0 | 3 (—) |
| Total | 381 (*100*) | 230 (*100*) | 80 (*100*) | 50 | 741 (*100*) |

remainder were recorded as being due to diseases of old age. However, all deaths regardless of cause have been included in the calculation of the survival rates, this being considered to provide the most objective assessment. The tables give rates at 5 years. The statistical tests are based on the difference between the rates divided by the standard error of the difference as calculated by Greenwood's (1926) method.

*Age and sex*

Disease-free and survival rates of patients by age are given in Table IV. It will be seen that patients over the age of 70 have a significantly poorer survival than the younger age groups but Table IV shows that the disease itself is not responsible for the greater attrition as the disease-free interval is the same in all age groups. Males and females have similar overall disease-free survival rates.

*Height*

Patients with tumours ≦8 cm from the anal verge have a significantly poorer prognosis than those with higher tumours (Table V). Of 501 patients with tumours lying <8 cm from the anal verge, 35% were alive at 5-years, compared to 48% of the 299 patients with a cancer lying between 8 and 15 cm. There was, however, no significant difference in the proportion of patients having curative resections, being 62% for patients with low-lying lesions (<8 cm from the anal verge) compared to 65% in patients with cancer in the upper rectum or recto-sigmoid. The 5-year local disease-free rate is different for lesions above and below 8 cms from the anal verge, being 62% and 52% respectively. Similarly, the disease-free survival rate at 5-years is considerably better (40%) for patients with higher level cancers than the 29% rate in those with lesions within 8 cms of the anal verge (Table V).

**Table IV** Age related to prognosis

| | | Age (yrs) | | | | |
|---|---|---|---|---|---|---|
| | | <60 (n=252) | 60–69 (n=300) | >70 (n=270) | $\chi^2$ | P |
| Curative resection rate (%(s.e.)) | | 66 (3) | 62 (3) | 61 (3) | 1.33 | 0.52 |
| Local disease-free rate (%(s.e.)) | 5 yr | 56 (3) | 58 (3) | 51 (3) | 2.44 | 0.30 |
| Survival rate (%(s.e.))[a] | 5 yr | 45 (3) | 44 (3) | 31 (3) | 14.1 | <0.001 |
| Disease-free survival rate (%(s.e.)) | 5 yr | 37 (3) | 35 (3) | 27 (3) | 7.85 | 0.02 |

[a]actuarial figures: not age corrected

**Table V** Height of tumour related to prognosis

| | | Height of tumour | | | |
|---|---|---|---|---|---|
| | | ≦8 cm (n=501) | >8 cm (n=299) | $\chi^2$ | P |
| Curative resection rate (%(s.e.)) | | 62 (2) | 65 (3) | 0.35 | 0.5 |
| Local disease-free rate (%(s.e.)) | 5 yr | 52 (2) | 62 (3) | 6.87 | 0.008 |
| Survival rate (%(s.e.)) | 5 yr | 35 (2) | 48 (3) | 12.9 | <0.001 |
| Disease-free survival rate (%(s.e.)) | 5 yr | 29 (2) | 40 (3) | 10.1 | 0.001 |

*Mobility*

The mobility of the cancer was seen to be of great prognostic importance. In 401 patients with mobile lesions 80% were considered to have had curative resections compared with only 44% of 364 patients who had tethered lesions. The 5-year local disease-free rate was greater in patients with mobile lesions (70%) compared to those with tethered cancers (37%). No great difference in prognosis was seen between patients with partially fixed and those with completely fixed tumours. For example, (Table VI) the 5-year survival rates were 30% and 25% respectively ($\chi^2=0.74$ on 1 df, $P=0.39$). However, the 5-year survival rate for the 401 mobile tumours was 48% compared with 29% for the 364 tethered tumours (Table VI) ($\chi^2=29.1$ on 1 df, $P<0.001$), and Figure 4.

*Quadrants involved*

Three hundred and ninty-four patients (48% of the total) were assessed as having only one quadrant involved. The prognosis is significantly better if one quadrant is involved. Curative resection was carried out significantly more often than when ≧2 quadrants were involved; 72% of the 394 patients with one quadrant involved had a curative resection compared with 55% of 399 patients with ≧2 quadrants involved. Quadrant involvement similarly influenced the disease-free rate, the survival rate and disease-free survival rate (Table VII). No information on the number of quadrants involved was available for 30 patients; their 5-year survival rate was 36%.

*Type of operation*

The type of operation performed appears to have comparatively little relationship to the prognosis in this series. The proportion of patients undergoing curative resection is similar (68% and 73%) after both types of operation, ($\chi^2=1.6$ on 1 df, $P=0.21$). There is also no significant difference in the local disease-free rates at 5 years (59% and 66%) ($\chi^2=2.0$ on 1 df, $P=0.16$). There is however an indication that the 5-year survival rate (41% and 55%) ($\chi^2=6.2$ on 1 df, $P=0.01$) and the disease-free survival rate (34% and 44%) ($\chi^2=5.1$ on 1 df, $P=0.02$) may be better in patients who were managed by anterior restorative resection.

*Curative resection*

Curative resection as earlier defined was found to be an extremely important factor in prognosis. In 519 patients who were considered to have undergone curative resection, the five year survival rate was 56% compared to only 13% of 304 patients who had residual disease at the time of operation ($\chi^2=209.7$ on 1 df, $P<0.001$). It has already been noted above that the mobility of the tumours significantly affects the number of curative sections performed.

**Figure 4** Mobility of tumour related to disease-free survival.

**Table VI** Mobility of tumour related to prognosis

| | | *Fixity of tumour* | | | |
|---|---|---|---|---|---|
| | | *Mobile* ($n=401$) | *Tethered* ($n=364$) | $\chi^2$ | *P* |
| Curative resection rate (%(s.e.)) | | 80 (2) | 44 (3) | 117.1 | <0.001 |
| Local disease-free rate (%(s.e.)) | 5 yr | 70 (2) | 37 (3) | 80.5 | <0.001 |
| Survival rate (%(s.e.)) | 5 yr | 48 (3) | 29 (2) | 29.1 | <0.001 |
| Disease-free survival rate (%(s.e.)) | 5 yr | 41 (3) | 23 (2) | 27.1 | <0.001 |

**Table VII** Number of quadrants involved related to prognosis

| | | *Number of quadrants involved* | | | |
|---|---|---|---|---|---|
| | | 1 ($n=394$) | 2–4 ($n=399$) | $\chi^2$ | *P* |
| *Curative resection rate* (%(s.e.)) | | 72 (2) | 55 (2) | 27.8 | <0.001 |
| Local disease-free rate (%(s.e.)) | 5 yr | 64 (3) | 48 (3) | 18.7 | <0.001 |
| Survival rate (%(s.e.)) | 5 yr | 47 (3) | 33 (2) | 16.7 | <0.001 |
| Disease-free survival rate (%(s.e.)) | 5 yr | 41 (3) | 26 (2) | 19.0 | <0.001 |

*Dukes' stage*

The probability of performing a "curative" resection was correlated with the Dukes' stage 91% of Dukes' A, 66% of Dukes' B and 58% of Dukes' C had a curative resection ($\chi^2 = 100.0$ on 2 df, $P < 0.001$). The local disease-free rate is also related to Dukes' staging, being 82% at 5 years for patients with Dukes' Stage A, 63% for Stage B tumours and 42% for those with Stage C tumours ($\chi^2 = 77.9$ on 2 df, $P < 0.001$). This parallels the highly significant differences in the survival (70%, 47% and 25%) ($\chi^2 = 108.8$ on 2 df, $P \leqq 0.001$) and the disease-free survival rates (65%, 37% and 20%) ($\chi^2 = 107.5$ on 2 df, $P < 0.001$) of patients at 5-years in the Dukes' Stages A, B and C.

*Histological grade*

The histological grade of the cancer was recorded in 727 (98%) of 741 patients who had their operative specimen examined. There is an important difference in the 5-year local disease-free rate which is 69% for patients with low grade tumours, 62% for average grade and only 42% in the group of 111 patients with high grade lesions ($\chi^2 = 17.1$ on 2 df, $P < 0.001$). The patients with low grade tumours had a 55% five year survival rate, whereas 45% of the patients with an average histological grading survived 5 years, as did 25% of the patients with high grade cancers ($\chi^2 = 25.2$ on 2 df, $P < 0.001$). The disease-free survival is also strongly related to the histological grade of the tumour. A greater proportion of patients (51%) with low grade tumours than patients with average (36%) or high grade lesions (21%) were alive and free of disease at 5 years ($\chi^2 = 26.8$ on 2 df, $P < 0.001$).

### Discussion

The clinico-pathological features described in this paper have been extracted from the data of 824 patients recruited to the First MRC Trial of Pre-operative Radiotherapy in Operable Rectal Cancer (1982), who were treated in 17 centres in the United Kingdom.

In making the comparisons recorded in this paper it should be remembered not only that two-thirds of the patients had been given radiotherapy, but that the group of patients treated in this way had similar outcomes, curative resection rates, local disease-free rates and survival rates to the control group (Second MRC report, 1984). The protocol of this trial was designed specifically to recruit patients with operable cancers of the rectum and this may explain why the percentage of Dukes' A cases is higher than in other reported series. Yet only 49% of patients had completely mobile tumours on pre-treatment assessment. Another 30% of patients were reckoned to have partially fixed cancers also considered suitable for radical surgery and 15% of patients had fixed cancers at pre-treatment assessment, but were still considered operable. It is important to note that two-thirds of the patients with tethered tumours received radical surgery.

Patients eligible for admission to this trial had an operable adenocarcinoma of the rectum with lower margin within 15 cm from the anal verge. Since the mean diameter of the tumours was 4.7 cm this corresponds approximately to tumours whose centre is up to 17–18 cm from the anal verge. The majority of patients (68%) were managed by abdomino-perineal excision and only 21% of patients were considered suitable for anterior restorative resection. This reflects the fact that the trial began in 1975 and therefore antedated the stapling era in which lower resection and anastomosis have become more feasible for the general surgeon. It must also be considered whether surgeons refrained from anastomosing bowel in radiation-treated patients because of the risk of fistula. In the first report of the MRC trial of radiotherapy in operable cancer of the rectum the incidence of fistula was higher in the control group than in the irradiated patients. The survival rates for anterior restorative resection are usually reported to be better than those following abdomino-perineal excision of the rectum (Mayo *et al.*, 1958; Deddish & Stearns 1961; Lockhart-Mummery *et al.*, 1976) as was demonstrated, though not very significantly, in this trial. The relationship to prognosis of the operation performed may have been reduced because of the large numbers of surgeons involved in the trial. The entry criteria to the trial were however restricted to patients with rectal cancer only. If those patients thought to have a better prognosis and suitable for a sphincter-conserving operation in the upper rectum, i.e. tumours of the rectosigmoid and sigmoid, were added the advantage to the group receiving anterior restorative operation might have been further magnified. It should not, however be concluded that the operation is responsible for the improved prognosis, since tumours higher in the rectum may from their natural history be less lethal. Relating to this it was found that tumours were assessed to be mobile more frequently in the upper part of the rectum.

The operability rate in the series was 90% and of these 69% were considered to have had a curative resection, figures which are consistent with other published series (Lockhart-Mummery *et al.*, 1976). The surgeons' assessment in this series that a curative resection had been performed agreed closely with the long-term prognosis.

The overall survival and disease-free survival rates were found to be strongly related to features determined at pre-treatment assessment; the single most important of which was the mobility of the primary cancer. Fixity of the tumour significantly reduced the probability of achieving a curative resection. This was subsequently reflected in a poorer disease-free survival in this group compared with patients who had mobile tumours. Evidence of local extension in relation to fixity, more than one quadrant involved and Dukes' Stage C generally indicated a poor prognosis as had been emphasised in other reports (Wood *et al.*, 1981).

Yet a Dukes' C case could be either fixed or mobile, while the corollary was established that small tumours, less invasive through the wall, could readily have lymph node metastases. It is therefore not surprising that many mobile tumours turned out to be Dukes' C stage. More surprising are the 12 instances of clinically fixed tumours which were reported as Dukes' A stage (Table III). One has to conclude that mobility may not always be accurately assessed by the surgeon for a number of reasons, among which may be included a wrong clinical appraisal but also surrounding inflammatory reaction. Nevertheless, despite the accepted margin of error in this assessment, it remains an important pre-operative prognostic factor (Figure 4).

Patients over the age of 70 years had a worse prognosis than younger patients. It was also found that patients with cancer arising >8 cm from the anal verge had a better disease-free survival rate than patients with lower lying cancers, although the curative resection rates were similar. Patients with only one quadrant of the rectum involved had a higher rate of curative resections and a higher disease-free survival rate than those with more extensive cancers. In view of the importance of these factors, all of which may be assessed pre-operatively, it would now seem possible to devise a method of clinical prognostic staging for patients with rectal cancer. Zorzitto *et al.* (1982) have suggested one such system. They do not include all the factors which we have found to relate to prognosis although their results agree with ours in that mobility of the tumour is found to be an assessment of primary importance. A clinical staging system would be useful in delineating those patients for whom adjuvant therapy might be considered, particularly if the adjuvant therapy were to be given pre-operatively. Dukes' staging, the histological grade of the cancer (Mayo *et al.*, 1958) and the surgeons' assessment of a curative operation were all very strongly related to the prognosis, as is well documented in the literature but can only be used post-surgery. The analysis presented here has identified factors which individually relate to prognosis. Many of these factors are inter-related and the independent effects of each factor on prognosis need to be disentangled. Multivariate analysis of the data may help to achieve this and if so should form the basis of sound clinical and clinico-pathological staging systems for rectal cancer.

We wish to thank Mary Stone, previously of the MRC Statistical Research and Services Unit, Bethan Smith of the MRC Cancer Trials Office for co-ordinating the data collection and Petra Macaskill of the MRC Cancer Trials Office for computer programming. The surgeons radiation oncologists and pathologists are as listed in the First report of an MRC Working Party (1982).

### References

DEDDISH, M.R. & STEARNS, M.W. (1961). Anterior resection for carcinoma of the rectum and rectosigmoid. *Ann. Surg.*, **154**, 961.

DUKES, C.E. (1940). Cancer of the rectum: An analysis of 1,000 cases. *J. Pathol. Bacteriol*, **50**, 527.

FIRST REPORT OF AN MRC WORKING PARTY (1982). A trial of pre-operative radiotherapy in the management of operable rectal cancer. *Br. J. Surg.*, **69**, 513.

GREENWOOD, M. (1926). *The Natural Duration of Cancer. Rep. Publ. Hlth. Med. Subj. No. 33*, London, H.M.S.O.

LOCKHART-MUMMERY, H.E., RITCHIE, J.K. & HAWLEY, P.R. (1976). The results of surgical treatment of carcinoma of the rectum at St. Mark's Hospital from 1948 to 1977. *Br. J. Surg.*, **63**, 673.

MAYO, C.W., LABERAGE, M.Y. & HARDY, W.M. (1958). Five year survival after anterior resection for carcinoma of the rectum and rectosigmoid. *Surg., Gynaecol. Obstet.*, **106**, 695.

NICHOLLS, R.J. (1982). *Recent Results in Cancer Research: Colorectal Cancer.* (Ed. Duncan), New York, Springer Verlag p. 101.

RIDER, W.D., PALMER, J.A., MAHONEY, L.J. & ROBERTSON, C.T. (1977). Pre-operative irradiation in operable cancer of the rectum: report of the Toronto Trial. *Can. J. Surg.*, **20**, 355.

ROSWIT, B., HIGGINS, G.A. Jr. & KEEHN, R.J. (1975). Pre-operative irradiation for carcinoma of the rectum and recto-sigmoid colon – report of the National Veterans' Administration randomised study. *Cancer*, **35**, 1597.

SECOND REPORT OF AN MRC WORKING PARTY (1984). The evaluation of low dose pre-operative X-ray therapy in the management of operable rectal cancer. Results of a randomly controlled trial. *Br. J. Surg.*, **71**, 21.

WOOD, C.B., GILLIS, C.R., HOLE, D., MALCOLM, A.J.H. & BLUMGART, L.H. (1981). Local tumour invasion as a prognostic factor in colorectal cancer. *Br. J. Surg.*, **68**, 326.

ZORZITTO, M., GERMANSON, T., CUMMINGS, B. & BOYD, N.F. (1982). A method of clinical prognostic staging for patients with rectal cancer. *Dis. Colon Rectum*, **25**, 59.